# Meteorological Table

**Published:** 1813-09

**Authors:** 


### METEOROLOGICAL TABLE.

*From July 25, to August 25, 1813.*

| | D. | Therm. | | | Barom. | | Hygrom. *Dry.——Damp.* | | | Winds | Atmos. Variation. |
|---|---|---|---|---|---|---|---|---|---|---|---|
| | 26 | 60 | 66 | 58 | $29^{6}$ | $29^{8}$ | 10 | 12 | 7 | W.. | F.. R.... F.. |
| 🌑 | 27 | 63 | 69 | 60 | $29^{9}$ | 30 | 10 | 13 | 5 | W. | F.. R.. F.. |
| | 28 | 63 | 76 | 64 | $30^{2}$ | — | 20 | — | — | NW. | F... |
| | 29 | 64 | 76 | 64 | $30^{3}$ | $^{2}$ | 15 | 30 | 22 | S.E. | F... —....—.... |
| | 30 | 64 | 79 | 76 | 30 | $29^{9}$ | 15 | 30 | 25 | S.SW. | F.... —.... —.... |
| | 31 | 66 | 73 | 68 | 30 | $30^{1}$ | 22 | 24 | 28 | NW. | F... F.. — ... C. |
| | 1 | 64 | 70 | 67 | $30^{1}$ | — | 20 | — | 19 | W.. | F. C.. F. |
| | 2 | 63 | 70 | 64 | 30 | $29^{9}$ | 15 | 20 | 15 | W. | R. C. F.. |
| | 3 | 63 | 69 | 63 | $29^{9}$ | $^{8}$ | 10 | 26 | 13 | W... | R. F.. R. F... |
| ☽ | 4 | 65 | 67 | 63 | $29^{9}$ | — | 13 | 35 | 22 | W... | F... F.. F.... |
| | 5 | 61 | 65 | 58 | $29^{7}$ | $^{5}$ | 16 | 23 | 8 | W... | R.. — .. F. R... |
| | 6 | 59 | 69 | 58 | $29^{6}$ | $^{9}$ | 6 | 11 | 10 | NW.. | F... — ... R. |
| | 7 | 58 | 70 | 67 | $29^{9}$ | 30 | 11 | 35 | 30 | NW. | F...— .... —.... |
| | 8 | 64 | 69 | 65 | 30 | — | 19 | 20 | 10 | W. | C.. F. R.. |
| | 9 | 60 | 69 | 65 | 30 | $30^{2}$ | 12 | 30 | 22 | N.. | F.. — .... —.... |
| | 10 | 61 | 68 | 65 | $30^{2}$ | — | 16 | 21 | — | N. | F... — .. R. F.... |
| | 11 | 65 | 71 | 69 | $30^{2}$ | $^{1}$ | 16 | 25 | 20 | SW. | C.. F... C.. F.... |
| ○ | 12 | 65 | 76 | 70 | 30 | — | 19 | 45 | 40 | W. | F.... — .... —.... |
| | 13 | 65 | 70 | 63 | $30^{1}$ | $^{2}$ | 19 | 40 | 30 | N.. | C..R..F....—.... |
| | 14 | 59 | 68 | 63 | $30^{2}$ | 30 | 21 | 35 | 22 | W. | F...C.. F... |
| | 15 | 63 | 70 | 64 | $29^{9}$ | — | 10 | 46 | 35 | NW.. | C.. F... — ... |
| | 16 | 62 | 66 | 62 | $29^{9}$ | — | 24 | 37 | 22 | N.. | C.—...F..—... |
| | 17 | 60 | 68 | 62 | $29^{9}$ | — | 22 | 30 | 22 | NW.. | C...F..C...F.... |
| | 18 | 60 | 70 | 64 | $29^{9}$ | 30 | 14 | 26 | 20 | W..N. | F.. — .. R.. |
| ☾ | 19 | 58 | 68 | 63 | $30^{1}$ | $^{2}$ | 20 | 45 | 37 | N.. | F... — .... — .... |
| | 20 | 61 | 68 | 59 | $30^{2}$ | — | 27 | 43 | 38 | NW. | F....— ... C.. F.... |
| | 21 | 55 | 63 | 57 | $30^{2}$ | $^{1}$ | 28 | 43 | 35 | NW.. | F... C.. F... |
| | 22 | 57 | 60 | 53 | $29^{9}$ | $^{8}$ | 27 | 20 | 21 | NW.. | F.. R.. —.. |
| | 23 | 55 | 64 | 59 | 30 | $30^{2}$ | 19 | 40 | 28 | N.. | F.. F.. — ... |
| | 24 | 57 | 64 | 62 | $30^{3}$ | — | 21 | 36 | 30 | N.NE. | F....—....—.... |
| | 25 | 58 | 65 | 60 | $30^{3}$ | — | 25 | 35 | 30 | E.NE. | F... —....—.... |

Quantity of rain from the 25th of July to the 25th of August, $\frac{62}{100}$ of an inch.

Serenity and mildness of temperature, and a dry atmosphere, have generally characterised this interval; but to this there has been some exceptions. On the 26th of July, there was a storm of thunder with heavy showers; the 21st of August was unpleasantly cold, morning and evening; and the 22d had a winterly aspect throughout, with stormy showers from the N.W.

The range of the thermometer and barometer has been unusually great in this interval. On the 30th of July, the temperature, at two o'clock P.M. was 79°; on the 22d of August, at two o'clock P.M. it was 60°: the heat has been below the standard of this season of the year. The barometer has varied also very considerably, and sometimes very suddenly. On the 5th of August in the evening, wind blowing fresh from W. the mercury was $29\frac{5}{10}$; on the 24th in the morning, wind nearly calm from N. $30\frac{3}{10}$. The winterly day on the 22d, barometer in the evening $29\frac{8}{10}$, wind blowing gently from N.W. On the morning of the 24th, the mercury had risen to $30\frac{3}{10}$, wind due N. with a brilliantly clear atmosphere, continuing to the evening of the 25th, when the whole visual hemisphere from Loudon was obscured, by congregated cirro-cumulus.

A most

A most seasonable and advantageous absence of atmospherical humidity has marked this period; the quantity of rain being little more than half an inch. It will be seen, by turning back to our Journal for September 1812, page 255, that the quantity of rain which fell from the 26th of July to the 26th of August of that year, was two inches and $\frac{41}{100}$, nearly four-fifths more than has fallen in the present August. From the 26th of July to the 26th of August 1812, it rained on fourteen days, and in large quantity on each day. From the 25th of July to the 25th of August 1813, it has rained eleven days, but most frequently in light and transient showers.

According to our own observation, the disease of the season, *Cholera*, has prevailed much more extensively in the present than in the autumn of 1812, showing, as far as this fact goes, that the production of this peculiar state of morbid action in the prima via, is not promoted by a humid atmosphere. Some cases of Rubeola, neither of the mildest or most severe form, have occurred; as have some of Varicella.

*Prince's Street, Cavendish Square.*

